# Development of Entrustable Professional Activities for entry into residency at the Charité Berlin

**DOI:** 10.3205/zma001213

**Published:** 2019-02-15

**Authors:** Ylva Holzhausen, Asja Maaz, Anna Renz, Josefin Bosch, Harm Peters

**Affiliations:** 1 Charité – Universitätsmedizin Berlin, Prodekanat für Studium und Lehre, Dieter Scheffner Fachzentrum für medizinische Hochschullehre und Ausbildungsforschung, Berlin, Germany

**Keywords:** Entrustable Professional Activities, curriculum development, undergraduate medical education, consensus methods

## Abstract

**Background: **Entrustable Professional Activities (EPAs) have emerged as a new approach to operationalise the workplace performance expectations for the transition from under- to postgraduate medical training. However, the transferability of such EPAs from one context to another appears limited. In this article, we report on the results of our approach to define a full set of core EPAs for entry into residency with the expectation to be performed under distant supervision.

**Methods: **The EPA development involved a modified, three round Delphi study, conducted at the Charité – Universitätsmedizin Berlin. The supervision level was operationalised as supervisor being distantly available and findings being reviewed. The threshold for consent was reaching a content validity index of a least 80%. The Delphi study involved experienced physicians (n=45) and resulted in a set of core EPAs with the descriptions of the categories: title, specification/limitations, conditions and implications of entrustment decision, knowledge, skills, and attitude, link to competencies and assessment sources.

**Results: **The response rates were 76-80% in the Delphi rounds. Key to the content validation process for the performance expectation was deciding on “to act under distant supervision”. The results are full descriptions of 12 core EPAs, organised into 5 overarching EPA domains.

**Conclusions: **Our systematic approach yielded the definition of 12 core EPAs for entry into residency at the level of “act with distant supervision” according to the practice in our context. This report may support other medical schools who plan to implement EPAs into their curricula.

## Zusammenfassung

**Hintergrund: **Entrustable Professional Activities (EPAs) haben sich als ein neues Konzept etabliert, um die Erwartungen an ärztliche Berufsanfänger am Arbeitsplatz zu operationalisieren. Die Übertragbarkeit von EPAs von einem auf den anderen Kontext ist nur begrenzt möglich. In diesem Artikel berichten wir über die Ergebnisse unseres Vorgehens, ein komplettes Set an Kern-EPAs zu entwickeln, die ärztliche Berufsanfänger unter entfernter Aufsicht ausführen können sollten.

**Methode: **Die Entwicklung der EPAs erfolgte mittels einer modifizierten, aus 3 Runden bestehenden Delphi-Studie an der Charité - Universitätsmedizin, Berlin. Als Aufsichtsgrad wurde festgelegt, dass supervidierende Ärzte mit zeitlicher Verzögerung zur Verfügung stehen und Wichtiges nachgeprüft wird. Ein Content Validity Index von mindestens 80% wurde als Grenze für den Konsens festgelegt. Die Delphi-Studie wurde mit erfahrenen Ärzten durchgeführt (n=45) und resultierte in ein Set von EPAs, die jeweils folgende Kategorien beinhalten: Titel; Spezifikationen/Limitationen; Bedeutung der Entrustment-Entscheidung; Kenntnisse, Fertigkeiten und Haltungen; Kompetenzbereiche und Assessment.

**Ergebnisse: **Der Rücklauf in den Delphi-Runden lag zwischen 76-80%. Voraussetzung für den inhaltlichen Validierungsprozess der Kerntätigkeiten war die Festlegung des Supervisionslevels auf „unter entfernter Aufsicht handeln“. Die Ergebnisse sind vollständige Beschreibungen von 12 EPAs, eingeordnet in 5 übergeordnete EPA-Domänen.

**Schlussfolgerungen:** Unser systematisches Vorgehen ergab die Definition von 12 Kern-EPAs für ärztliche Berufsanfänger mit dem Aufsichtsgrad „unter entfernter Aufsicht handeln“ für die Anwendung in unserem Kontext. Dieser Bericht soll andere medizinische Fakultäten unterstützen, die eine Implementierung von EPAs in ihre Curricula planen.

## 1. Background

The progress from undergraduate to postgraduate training represents an important and critical transition in medical education. This holds true for the institutions and educators involved, as well as for the individual trainee and future medical doctor. Competency-based medical education (CBME) has provided a shared conceptual framework for both undergraduate and postgraduate programs, and has thereby led to a better alignment of the two training periods [1], [2]. However, CBME does not translate well into real-world clinical practice [3]. There is nevertheless a need for a tangible operationalisation that defines the workplace performance expectations for residents when entering the postgraduate training, which in turn would represent the overarching outcomes that should be reached by graduates at the end of undergraduate education. The concept of Entrustable Professional Activities (EPAs) has emerged as a meaningful approach to specify educational outcomes in postgraduate medical training in several countries [4]. In Germany, the EPA has received an increasing amount of attention in postgraduate medical education [5], [6], [7]. Its application to the transition from undergraduate to postgraduate medical education is currently an active field of development and research [8], [9], [10], [11], [12], [13], [14]. Although examples of such core EPAs for entry into residency have become available, their application to other contexts and countries is limited. Many medical schools who consider the implementation of EPAs into their programs have to undergo their own EPA development process according to their context. Key to this process is specifying the supervision level at entry into residency. In this article, we report on the results of our approach to define a full set of core EPAs for entry into residency at the Charité – Universitätsmedizin Berlin (Charité) according to the medical practice of our educational setting.

The specification of educational outcomes by the EPA concept builds on two of its key elements:

1. professional activities which represent authentic workplace tasks characteristic of a profession, and
2. levels of supervisions, i.e. the degree of supervision a trainee needs to carry out a professional activity [3], [4].

The professional activities can range from smaller tasks for junior trainees to the full range of all tasks characteristic of a discipline being envisaged for senior physicians when completing their postgraduate medical training [4]. Over the course of medical training, the supervision levels gradually decrease from “allowed to observe only” (level 1) and “act under direct observation” (level 2) in early training, over “act under indirect supervision” (e.g. being available on request, level 3) to “act unsupervised” in later training stages (level 4) [15]. The combination of these two components – i.e. professional activities and supervision levels – can be employed to operationalise the performance-based outcomes in medical education. A further strength of this approach is that it builds on real-world workplace practices and uses a language that communicates well with medical supervisors, trainees and program directors. 

The content definition of educational outcomes by EPAs is generally achieved through a step-by-step consent process among content experts. It begins with the identification of authentic professional tasks, secondly the elaboration of its characteristics, followed by the content validation procedure [4], [16]. Key to the definition of EPAs for entry into residency is choosing and specifying a supervision level which reflects the supervision practice for graduating physicians in a particular context. Chen at al. have introduced a more granular operationalisation of supervision levels, especially for “act under indirect supervision” [8]. Closer or less close supervision will have an impact on the breadth and depth elaboration of entry into residency EPAs, for instance the spectrum of patient and disease complexity to be included. According to the current literature, an EPA definition should yield a seven-category description to fully define its content [4], [16], [17].

So far, core EPAs for entry into residency have been reported at a national level for the USA [9], Canada [10] and Switzerland [11]. While these sets of EPAs have several features in common, there are also tangible differences in the tasks which constitute the core of activities expected from all entering residents and the level of task difficulty to ensure they are manageable for entering residents. These differences likely reflect differences in the medical practice of these countries and indicate that one set of core EPAs for entry into residency cannot automatically be transferred from one context to another. All three EPA sets do not clearly specify the expected level of supervision at entry into residency. For the USA, the goal is without direct supervision (> level 2), for Canada it is with indirect supervision (supervisor not in the room but available to provide assistance) and for Switzerland it is distant, on-demand supervision (both level 3). The supervision levels leave open the temporal availability of the supervisor and how the findings and approaches of the resident are checked afterwards. We think this step is critical for the further content elaboration and validation of the breadth and depth of core EPAs for entry into residency in any context. The benefits of using a more granular supervisions levels as introduced by Chen at al. have shown in a recent article reporting on the definition of EPAs for the undergraduate medical curriculum in Utrecht, the Netherlands [12].

At the Charité, we have recently undergone a curricular reform of the undergraduate medical program [18]. The new curriculum was developed and implemented semester-by-semester in a faculty-wide approach on basis of a pre-defined outcome catalogue comparable to the Nationaler Kompetenzbasierter Lernzielkatalog (NKLM) and the CanMEDS framework [19], [20]. The result was a competency-based, vertically and horizontally integrated undergraduate medical curriculum with the first graduates entering postgraduate training in 2016. As professional activities served as the main longitudinal curriculum structure, there was a need to define EPAs as overarching outcomes for the program and for entry into residency [18]. In our context, the vast majority of graduating physicians start their postgraduate training in a hospital setting, where they mainly take care of adult patients. The general expectation is that the graduating physicians are in charge of adult patients on the ward during the day shift under distant supervision. The typical workflow of supervision encompasses daily routine meetings between trainee and supervising physician where new patients are presented and key medical decisions on all ward patients are coordinated. In addition, the trainee and supervisor jointly undertake full ward rounds twice a week. This aligns with supervision level 3c (supervisor distantly available and findings are reviewed) according to Chen at al. [8]. 

The aim of this article is to report on the results of a Delphi study defining a set of core EPAs delineated as full, seven-category descriptions. According to our context, they operationalise the clinical performance expectation for medical graduates entering residency, when acting under distant supervision. This is operationalised as the supervisor is not on the ward and findings are reviewed afterwards. 

## 2. Methods

### Setting

The study was conducted from January 2014 to November 2015 at the Charité. Its undergraduate medical program has an intake of more than 600 students per year, spans over six years and 5500 teaching hours in total. The Charité data protection office and ethics board approved the study (No EA2/091/14, Ethics Board Charité, Campus Mitte).

#### Delphi study

The approach was based on a modified, 3 round online Delphi consensus procedure. This process has been reported in detail elsewhere [21]. To summarise, it involved a step-by-step interaction between a writing team and a multidisciplinary panel of 45 purposely selected physicians from the Charité faculty body with long-time clinical supervision experience and active involvement in the curriculum development process of the current undergraduate medical program (e.g. as module board members or department teaching coordinators). 

The starting point was the identification of core tasks expected from all graduating physicians when entering into residency to be performed under distant supervision. During the course of the Delphi study, participants received the description of various EPAs and had to rate the relevance and the specific content of the presented categories on a 4-point Likert scale (disagree / somewhat disagree/ somewhat agree / agree) [21]. The threshold for consent was reaching a content validity index (CVI) of ≥80% [22]. CVI represents the percentage of respondents who agreed or somewhat agreed to the description of a certain EPA category. Descriptive statistics were calculated using IBM SPSS statistics 23 following each Delphi round, including mean (M) and standard deviation (SD). 

In Delphi round 1, panel members could propose EPAs not included in the initial list provided. During the Delphi procedure, panel members provided both quantitative ratings and qualitative narratives on the EPA categories until consent was achieved: title (round 1-2), specification/limitations (round 1-3), conditions and implications of entrustment decision (round 2-3), and knowledge, skills, and attitude (round 3). It has recently been described in detail how we decided on which categories to include in the respective Delphi rounds [21].

The writing team adjusted the EPA category descriptions on the basis of panel members´ ratings and comments. The EPAs were finally arranged according to the five overarching EPA domains to provide a more coherent picture [12].

## 3. Results

### Delphi response rate and panel members

The response rates were 80, 78 and 76 % in the three Delphi rounds with 36, 35 and 34 faculty members participating in each respectively. Thirty-two participants completed all three rounds. Participants’ demographics and characteristics are depicted in table 1 (Tab. 1). 

#### Identification of core EPAs for entry into residency

The panel members reached consensus on a total of 12 core activities with clinical performance expectations for entry into residency at the Charité. Table 2 (Tab. 2) displays the titles of the 12 core EPAs as grouped within 5 EPA domains. The panel members proposed an additional 20 topics for EPAs not listed in the initial list circulated in Delphi Round 1. A number of these suggestions could be integrated into the 12 EPAs, for instance “to document in the patient file” or “demonstrate sufficient understanding of basic science”. Others were of general nature, such as “show good time management”, or “recognise communication in stressful situations as a challenge”. The writing team decided that none of the proposed topics qualified as a separate EPA. 

#### Content elaboration and validation of core EPAs for entry into residency

During the Delphi process, the panel members reached consensus on the relevance ratings of the EPAs. Table 2 (Tab. 2) provides an overview of the ratings during the three Delphi rounds. The relevance rating indicates the match between the descriptions of an EPA with the expectation for entry into residency. In the case of a low CVI for an EPA, the EPA description was refined on the basis of the panel members´ narrative comments. In most cases the EPA specifications were perceived as too difficult, for entering residents to be able to perform them under distant supervision. In Delphi round 1, six EPA descriptions reached a CVI of 80% and higher, in round 2 a total of 10 EPA descriptions, and in round 3 all 12 EPA descriptions. Table 3 (Tab. 3) provides an overview of the panel member ratings during the Delphi process for the EPA category descriptions “title”, “specification/limitations”, “conditions and implications of entrustment decisions” and “knowledge, skills and attitude”.

Key to the content validation process was the elaboration and definition of “to act under distant supervision” in the EPA category “conditions and implications of entrustment decision”. This was operationalised as the trainee acting autonomously and his or her findings and decisions being reviewed by the supervisor during the next regular meeting or ward round. In the subsequent process, this level of autonomy led to a specification of the patients as adults, presenting with typical clinical presentations or common diseases and no major difficulties involved. In an attempt not to exclude some patient groups and medical disciplines, the limitation section of each EPA emphasises that a closer level of supervision is required and a lesser degree of autonomy is granted in the case of unstable or critically ill patients, new-borns, infants, children, pregnant women and discipline-specific clinical presentations or diseases. 

Table 4 (Tab. 4) provides the description of EPA 1 “Gather a medical history, perform a physical exam and provide a structured summary of the results”. The article appendix includes the full description of all 12 EPAs as developed in this Delphi study. In addition to information on the context, smaller tasks were incorporated to describe what is included in an EPA. This led to two types of nested EPAs: one, in which the EPA is specified by a chronological order of all activities included (EPA 1.1-1.4, 3.1, 4.2 and 5.2), and another, in which the EPA forms a collection of tasks from the same group (EPA 2, 3.2, 4.1, 4.3 and 5.1) (see attachment 1 ). 

## 4. Discussion

The present study reports on the results of a process of defining 12 core EPAs for entry into residency at the level of “act with distant supervision”. The EPA content was established in a step-by-step process based on a modified Delphi procedure and a systematic interaction between a multidisciplinary faculty expert panel and a writing team of educational experts [21]. The results of this process are full, seven-category descriptions of 12 EPAs which operationalise the performance expectations of graduating physicians according to the workplace practice and the new competency-based undergraduate medical program in our context at the Charité. In the following sections, we will discuss the results and implications of our study in the light of current literature.

In a modified Delphi study, content experts from our medical school were intentionally involved to make the EPAs as relevant as possible to our setting and to gain the support of those who will eventually work with them. Decisive for our EPA definition process was the designation of a supervision level for entry into residency. According to the practice in our context, we chose “to act with distant supervision” as an anchor to define the content description of our 12 core EPAs by our panel members. This designated supervision level was subsequently operationalised during the Delphi process when the panel members expressed uncertainty during the panel meetings and in their narrative feedbacks as to how this level of autonomy would actually translate into the supervision practice. Their uncertainty connects to literature on trust and EPAs noting that entrustment decisions require the specification of exactly what has been decided [23], [24], [25]. Entrustment relates to the acceptance that the trustee is permitted to act in circumstances where risks are present but still manageable [23]. To approach the panel members´ uncertainty, we specified for each EPA in detail when and to which degree the findings and decisions by the trainee are to be checked by the supervisor in the EPA category “conditions and implications of entrustment decision”. Our operationalisation aligns well with supervision level 3c by Chen and colleagues (supervisor not immediately available, findings are reviewed), which had not been published when we defined our supervision level [8].

Next, with “distant supervision” being set as the leading expectation, it became apparent that the difficulty of the tasks needed more specification to be manageable for graduating physicians. For this, we anchored the task complexity by referring to patients as adults with typical presentations, common diseases and typical courses of a disease. We can envision that in a specific clinical setting, this context can be further clarified according to the actual workplace practice. For instance, this could involve the 10-15 most common clinical presentations, diseases, drugs and procedures for which the distant supervision level is granted. To further curtail the EPA difficulty, we generally assigned a closer than distant supervision level for high-risk patients requiring urgent care, new-borns, children, pregnant woman and patients with special discipline-specific complaints and diseases.

The result of our modified Delphi study were 12 core EPAs, i.e. authentic units of work which we arranged for a more logical listing into 5 EPA domains as proposed by ten Cate et al. [12]. For instance, the four EPAs within the EPA domain “along the clinical encounter” can be seen as one workflow, but they represent separate EPAs, because a check by the supervisor is needed before the next EPA can be carried out. We identified 12 core EPAs, which is in the range of 9 to 13 EPAs identified by others [9], [10], [11], [12]. In addition, there is an overlap in the breadth and scope of these sets of core EPAs. However, there are also several differences. First, there are differences in splitting and arrangement of the tasks. For example, others have defined the EPA to “prioritize a differential diagnosis” [9], [11]. In our study, this task is integrated into EPA 1.1 “Gather a medical history, perform a physical exam and provide a structured summary of the results” and 1.2. “Compile a diagnostic work plan and initiate implementation” according to the workplace practice in our setting. Second, there are differences in the actual tasks chosen. For example, others have included an EPA for system improvements [9], [10], [11]. In our setting, there is no such activity in the workplace for entering residents, and thus this task is not part of our 12 EPAs. There are also differences in the number of medical procedures to be carried out by a graduating physician. According to practice in our setting, we incorporated 15 medical procedures, while others included only four [9]. Finally, we decided to organise our EPAs in such a manner that the specifications of the EPAs are described as nested tasks to make them observable and suitable for assessment purposes in early semesters. 

Our report holds several implications which go beyond the study itself. First, this study adds further example to the current search on how to define EPAs for entry into residency. It may serve as a stimulus and contribution for the definition of EPAs at a national level in Germany and the intended future development of the NKLM. Second, our Delphi study produced relevant faculty developments and ownership at our institution on how to translate CBME into practice by using the concepts of EPAs. Furthermore, the set of EPAs will be implemented as overarching outcomes for our undergraduate medical program with the intention to better prepare medical students for the workplace requirements in residency training. In order to reach this goal, the set of core EPAs should be employed to identify gaps, align learning sequences in a meaningful manner, adjust the assessment program and connect classroom and workplace learning in the undergraduate curriculum. Finally, the 12 core EPAs for entry into residency provide a blueprint for future educational research, for instance on the level of preparedness of graduates of our program on those core professional activities.

This work has limitations. With regard to the content expert panel, there may have been a selection bias as only a subgroup of the faculty was invited to take part. In addition, first year residents were not included but could have been an important source for content validation. Moreover, we did not operationalise the performance expectations for the closer supervision level in special patient groups. The generalizability of the resulting EPAs to settings with differences in workplace practice is limited.

## 5. Conclusions

In conclusion, this study reports on the results of defining 12 core EPAs for entry into residency at the level of “act with distant supervision” by Charité faculty members. This report aims to provide support and encouragement to other medical schools considering the implementation of EPAs in their curricula. 

## Acknowledgements

We would like to thank all participating faculty members for their valuable input, constructive feedback and exceptional commitment. We would also like to thank the WATCHME team for the discussions on the EPA concept and its application. The exchange substantially contributed to our process and results of developing EPAs.

## Funding

The study was funded as part of the initiative “Bologna – Zukunft der Lehre” by the foundation “Stiftung Mercator and Volkswagen Stiftung”; the “European Union’s Seventh Framework Programme for research, technological development and demonstration” under grant agreement 619349 (WATCHME Project); and the initiative “Hochschulpakt MSM 2.0 “Modellstudiengang Medizin 2.0” (01PL16036)” by the foundation “Bundesministerium für Bildung und Forschung”. 

## Competing interests

The authors declare that they have no competing interests. 

## Supplementary Material

Entrustable Professional Activities for entry into residency

## Figures and Tables

**Table 1:**
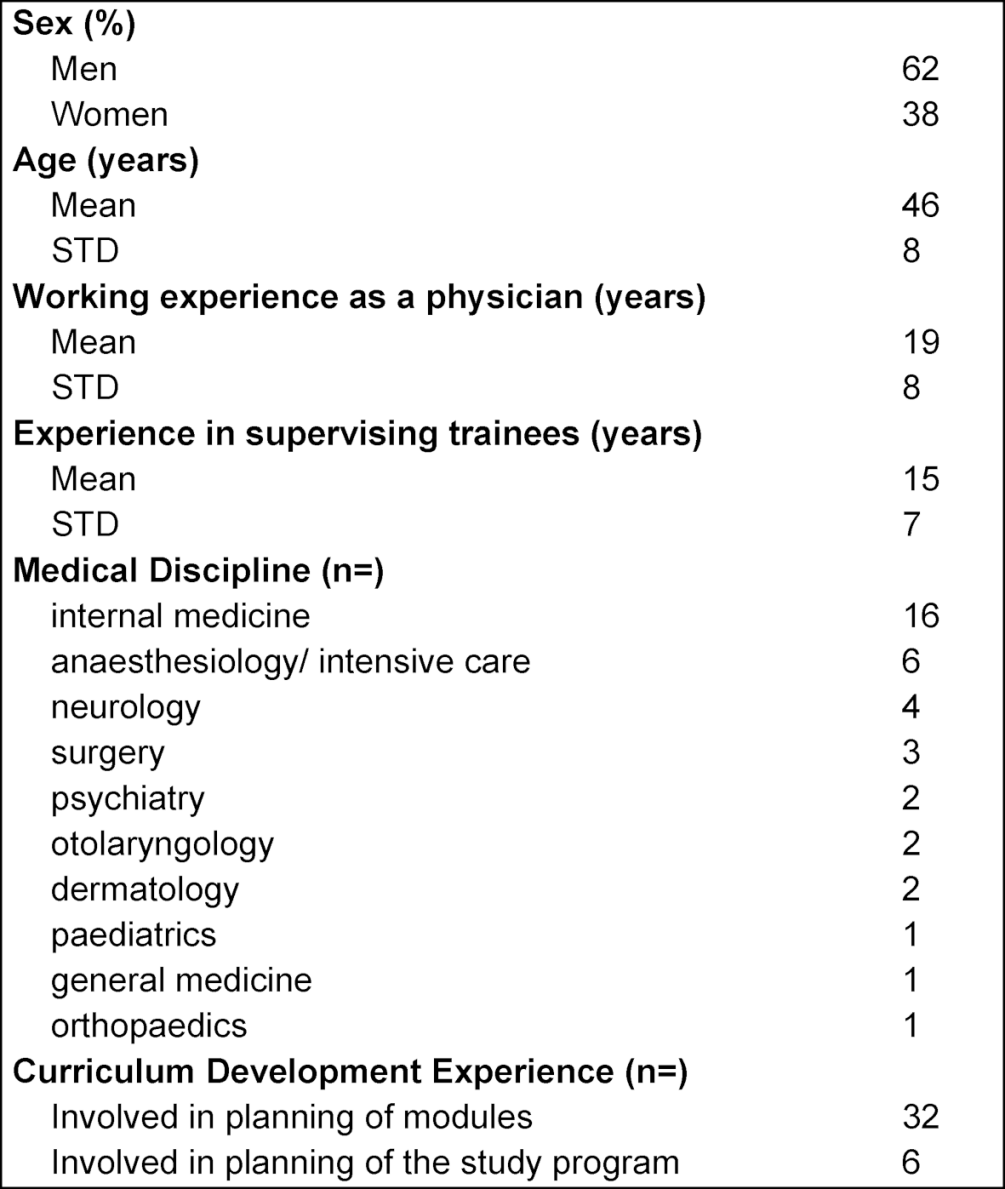
Participants’ demographics and characteristics

**Table 2:**
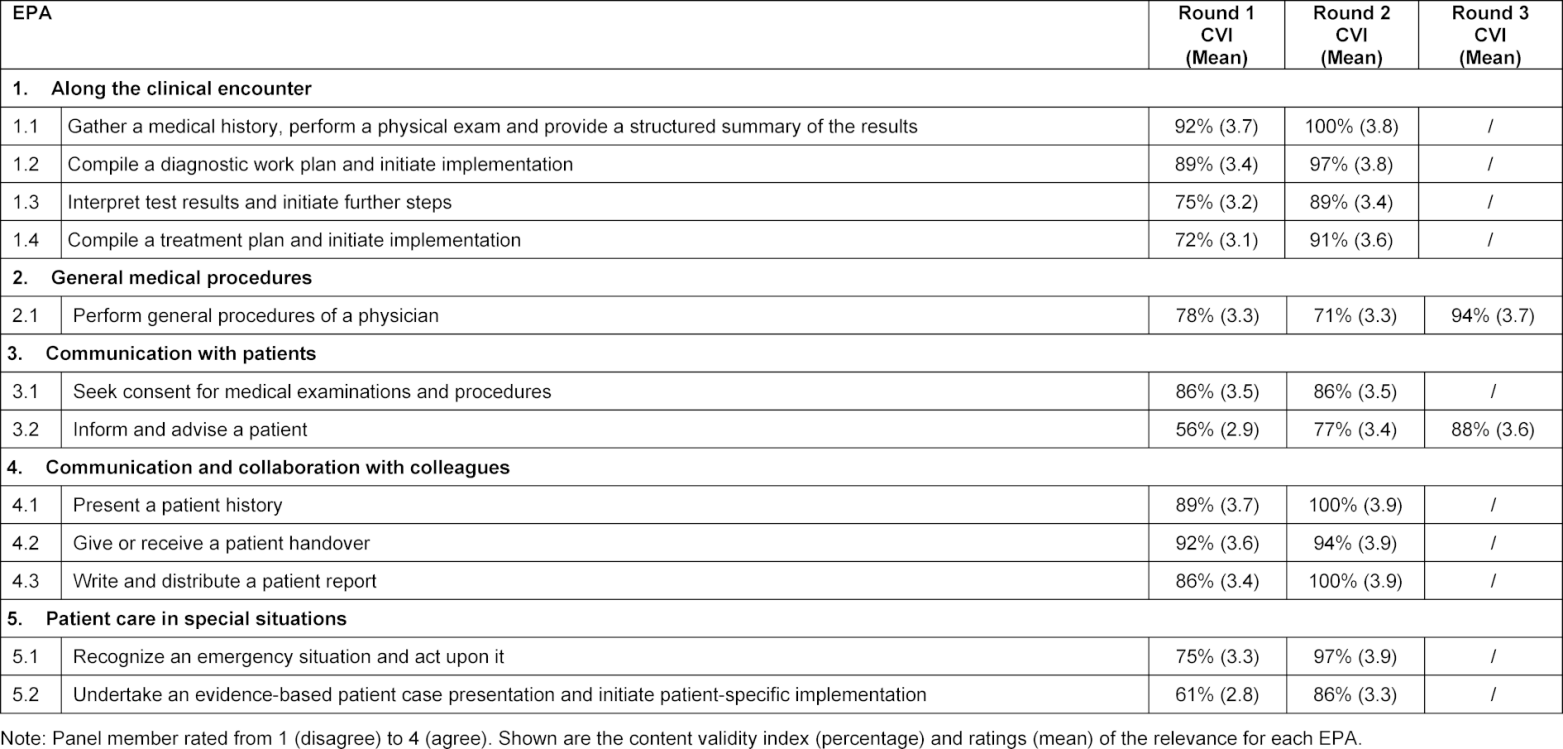
Titles and Content Validity Indexes (CVI) on the relevance of 12 core EPAs for entry into residency, grouped into 5 EPA domains.

**Table 3:**
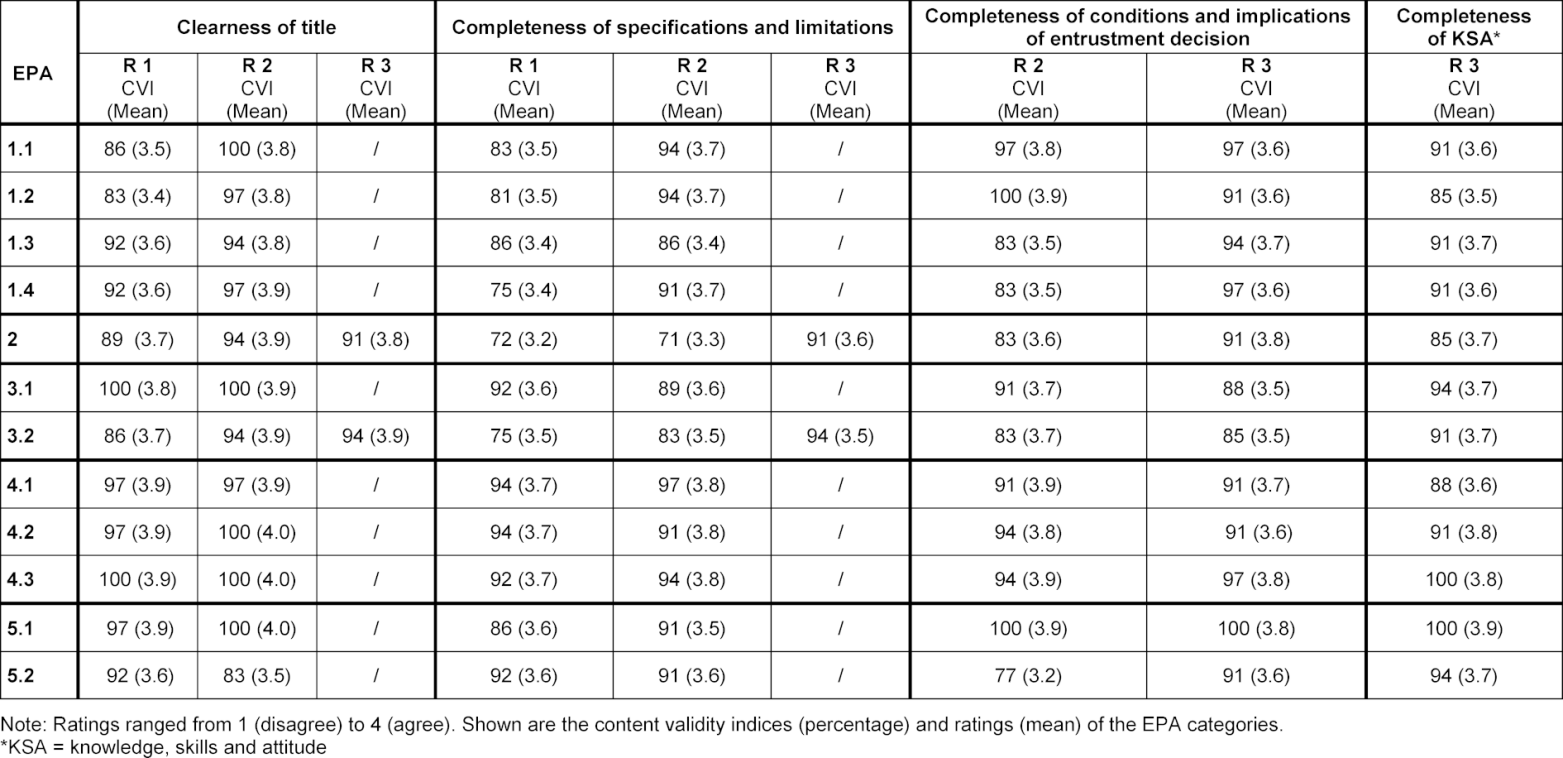
Results of panel member ratings in Delphi round 1 to 3 (R 1 to R 3) on the category descriptions of 12 core EPAs for entry into residency.

**Table 4:**
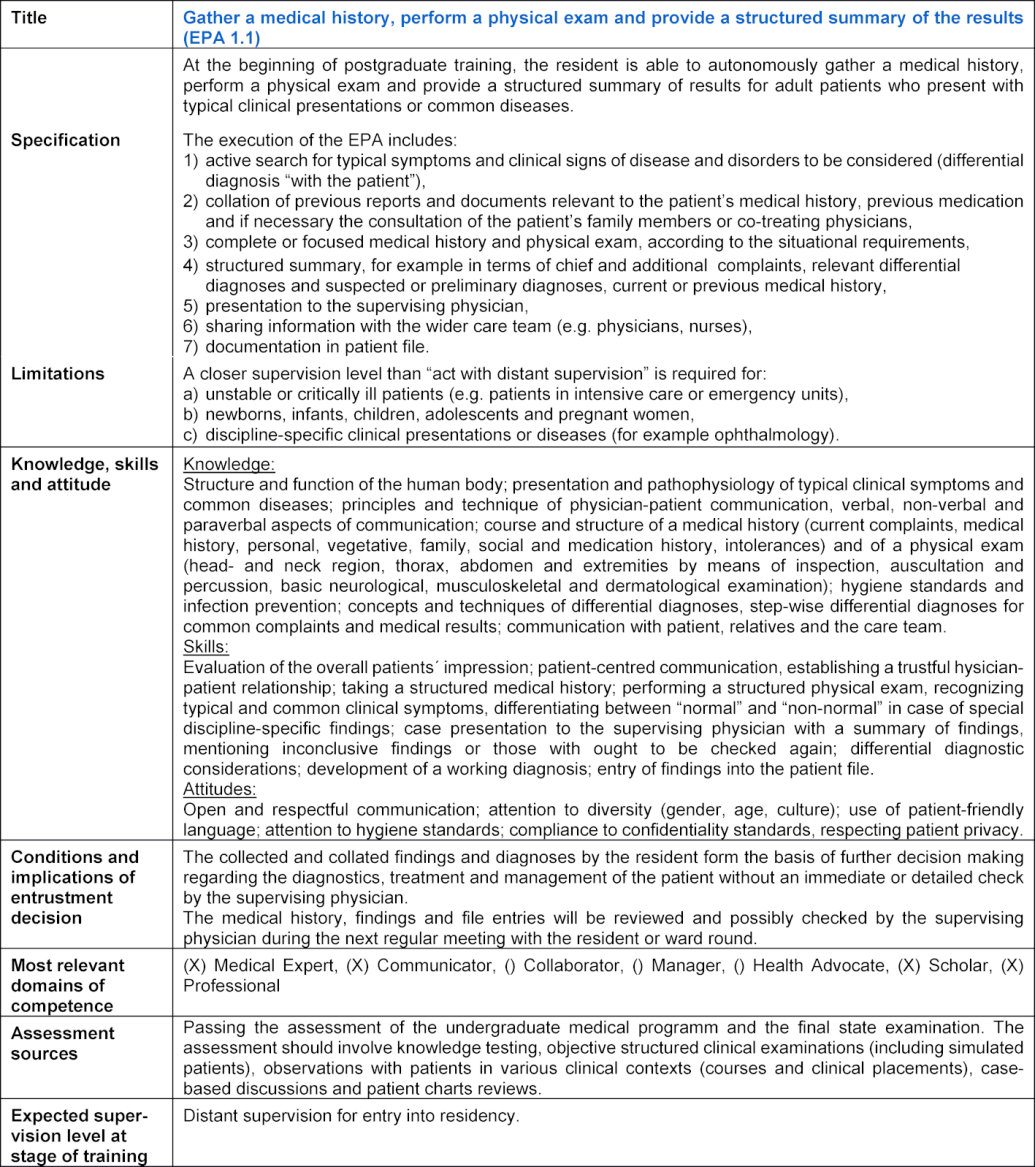
Gather a medical history, perform a physical exam and provide a structured summary of the results.
