# Supplementary material for: Development of Entrustable Professional Activities for entry into residency at the Charité Berlin
Source: GMS J Med Educ. 2019 Feb 15;36(1):Doc5. doi: 10.3205/zma001213 (PMC6390089; doi:10.3205/zma001213)
Supplement: Entrustable Professional Activities for entry into residency [file JME-36-5-s-001.pdf]

Appendix to the article:
Holzhausen Y, Maaz A, Renz A, Bosch J, Peters H. Development of Entrustable Professional Activities for entry into residency at the Charité Berlin. GMS J Med Educ. 2019;36(1):Doc5. DOI: 10.3205/zma001213, URN: urn:nbn:de:0183-zma0012137

English Version

Entrustable Professional Activities for entry into residency

**1.**	**Along the clinical encounter**
1.1	Gather a medical history, perform a physical exam and provide a structured summary of the results
1.2	Compile a diagnostic work plan and initiate implementation
1.3	Interpret test results and initiate further steps
1.4	Compile a treatment plan and initiate implementation
**2.**	**General medical procedures**
2.1	Perform general procedures of a physician
**3.**	**Communication with patients**
3.1	Seek consent for medical examinations and procedures
3.2	Inform and advise a patient
**4.**	**Communication and collaboration with colleagues**
4.1	Present a patient history
4.2	Give or receive a patient handover
4.3	Write and distribute a patient report
**5.**	**Patient care in special situations**
5.1	Recognize an emergency situation and act upon it
5.2	Undertake an evidence-based patient case presentation and initiate patient-specific implementation

EPA domain 1 – Along the clinical encounter

**Title**	**Gather a medical history, perform a physical exam and provide a structured summary of the results (EPA 1.1)**
**Specification**	At the beginning of postgraduate training, the resident is able to autonomously gather a medical history, perform a physical exam and provide a structured summary of results for adult patients who present with typical clinical presentations or common diseases.<br>The execution of the EPA includes:<br>1) active search for typical symptoms and clinical signs of disease and disorders to be considered (differential diagnosis “with the patient”),<br>2) collation of previous reports and documents relevant to the patient’s medical history, previous medication and if necessary the consultation of the patient’s family members or co-treating physicians,<br>3) complete or focused medical history and physical exam, according to the situational requirements,<br>4) structured summary, for example in terms of chief and additional complaints, relevant differential diagnoses and suspected or preliminary diagnoses, current or previous medical history,<br>5) presentation to the supervising physician,<br>6) sharing information with the wider care team (e.g. physicians, nurses),<br>7) documentation in patient file.
**Limitations**	A closer supervision level than “act with distant supervision” is required for:<br>a) unstable or critically ill patients (e.g. patients in intensive care or emergency units),<br>b) newborns, infants, children, adolescents and pregnant women,<br>c) discipline-specific clinical presentations or diseases (for example ophthalmology).
**Knowledge, skills and attitude**	Knowledge:<br>Structure and function of the human body; presentation and pathophysiology of typical clinical symptoms and common diseases; principles and technique of physician-patient communication, verbal, non-verbal and paraverbal aspects of communication; course and structure of a medical history (current complaints, medical history, personal, vegetative, family, social and medication history, intolerances) and of a physical exam (head- and neck region, thorax, abdomen and extremities by means of inspection, auscultation and percussion, basic neurological, musculoskeletal and dermatological examination); hygiene standards and infection prevention; concepts and techniques of differential diagnoses, step-wise differential diagnoses for common complaints and medical results; communication with patient, relatives and the care team.<br>Skills:<br>Evaluation of the overall patients´ impression; patient-centred communication, establishing a trustful physician-patient relationship; taking a structured medical history; performing a structured physical exam, recognizing typical and common clinical symptoms, differentiating between “normal” and “non-normal” in case of special discipline-specific findings; case presentation to the supervising physician with a summary of findings, mentioning inconclusive findings or those with ought to be checked again; differential diagnostic considerations; development of a working diagnosis; entry of findings into the patient file.<br>Attitudes:<br>Open and respectful communication; attention to diversity (gender, age, culture); use of patient-friendly language; attention to hygiene standards; compliance to confidentiality standards, respecting patient privacy.

**Conditions and implications of entrustment decision**	The collected and collated findings and diagnoses by the resident form the basis of further decision making regarding the diagnostics, treatment and management of the patient without an immediate or detailed check by the supervising physician.<br>The medical history, findings and file entries will be reviewed and possibly checked by the supervising physician during the next regular meeting with the resident or ward round.
**Most relevant domains of competence**	(X) Medical Expert, (X) Communicator, () Collaborator, () Manager, () Health Advocate, (X) Scholar, (X) Professional
**Assessment sources**	Passing the assessment of the undergraduate medical programm and the final state examination. The assessment should involve knowledge testing, objective structured clinical examinations (including simulated patients), observations with patients in various clinical contexts (courses and clinical placements) and case-based discussions.
**Expected super-vision level at stage of training**	Distant supervision for entry into residency.

**Title**	**Compile a diagnostic work plan and initiate implementation (EPA 1.2)**
**Specification**	At the beginning of postgraduate training, the resident is able to autonomously compile a step-wise diagnostic work-up plan for adult patients with typical clinical presentations, medical results or common diseases and to initiate its implementation following coordination with the supervising physician.<br>The execution of the EPA includes:<br>1) requesting basic diagnostic tests (i.e. monitoring weight, balances, blood pressure and pulse, basic blood readings, ECG, and thorax x-ray),<br>2) compiling a differential diagnostic work-up plan according to the symptoms, disease manifestation, medical results and patient preferences,<br>3) presenting the plan to the supervising physician and achieving agreement.<br>Following the coordination with the supervising physician:<br>4) requesting patient-specific diagnostics,<br>5) completing diagnostic order request forms,<br>6) sharing information with the patient and, where appropriate, with family members.<br>7) sharing information with the wider care team (e.g. physicians, nursing).
**Limitations**	A closer supervision level than "act with distant supervision" is required for:<br>a) unstable or critically ill patients (e.g. patients in intensive care or emergency units),<br>b) newborns, infants, children, adolescents and pregnant women,<br>c) discipline-specific clinical presentations or diseases (for example ophthalmology).
**Knowledge, skills and attitude**	Knowledge:<br>Pathophysiology of typical clinical symptoms and common diseases; concepts and techniques of differential diagnoses, step-wise differential diagnoses for common complaints and medical results; standards for basic diagnostic testing; standards or established diagnostic work-up for typical clinical symptoms and common diseases; course, benefits, risks, indications and contraindications of common diagnostic procedures (laboratory, imaging techniques with or without ionising radiation or radionuclides, endoscopic and electrophysiological procedures, pathology).<br>Skills:<br>Compiling a patient-specific, differential diagnostic work-up plan; consultation with supervisor; implementation of the patient-specific diagnostic work-up plan; rational medical decision-making; sharing information with the patient and care team.<br>Attitudes:<br>Attention to diversity (gender, age, culture); balancing effort, expense and risks of diagnostics with benefits and results; dealing with uncertainty.
**Conditions and implications of entrustment decision**	The resident autonomously requests basic and patient-specific diagnostics following coordination with the supervising physician. He/ she initiates the implementation without an immediate or detailed check by the supervising physician.<br>The implementation of the diagnostic work-up plan will be reviewed and possibly checked by the supervising physician during the next regular meeting with the resident or ward round.
**Most relevant domains of competence**	(X) Medical Expert, () Communicator, (X) Collaborator, (X) Manager, () Health Advocate, (X) Scholar, () Professional
**Assessment sources**	Passing the assessment of the undergraduate medical programm and the final state examination. The assessment should involve knowledge testing, objective structured clinical examinations (including simulated patients), observations with patients in various clinical contexts (courses and clinical placements) and case-based discussions.
**Expected super-vision level at stage of training**	Distant supervision for entry into residency.

**Title**	**Interpret test results and initiate further steps (EPA1.3)**
**Specification**	At the beginning of the postgraduate training, the resident is autonomously able to interpret results of common tests and to classify them generally for adult patients, recognizing common emergencies. He/ she initiates further steps following coordination with the supervising physician.<br>The execution of the EPA includes:<br>1) reviewing and interpreting findings of basic diagnostic tests (e.g. monitoring weight, balances, blood pressure and pulse, pulse oximetry, standard laboratory parameters, 12-lead ECG, chest X-ray),<br>2) reviewing and interpreting findings of common further examination methods (e.g. general laboratory parameters, microbiology, virology, endoscopy, radiological imaging),<br>3) recognising common emergency situations and acting upon them, (in accordance with EPA 12 “recognising emergencies and acting upon them”),<br>4) reporting to the supervising physician, and if necessary coordinating of subsequent diagnostic and therapeutic steps,<br>5) requesting and initialising further diagnostic tests and treatments,<br>6) sharing information with the patient and, where appropriate, with family members,<br>7) sharing information with the wider care team (e.g. physicians, nursing).
**Limitations**	A closer supervision level than “act with distant supervision” is required for:<br>a) unstable or critically ill patients (e.g. patients in intensive care or emergency units),<br>b) newborns, babies, children, adolescents, pregnant women,<br>c) discipline-specific clinical presentations or diseases (for example ophthalmology).
**Knowledge, skills and attitude**	<u>Knowledge:</u><br>Parameters to evaluate the significance of diagnostic results; implication of results from basic diagnostics and common further examination methods, including emergency situations.<br><u>Skills:</u><br>Reviewing and interpreting patient test results; adjusting, consultation with the supervising physician; implementation of the patient-specific diagnostic or treatment plan; rational medical decision-making; informing patients and the care team.<br><u>Attitudes:</u><br>Attention to diversity (gender, age, culture); balancing effort, expense and risks of diagnostics against benefits and results; dealing with diagnostic uncertainty.
**Conditions and implications of entrustment decision**	The resident’s report on the basic diagnostics and common tests form the basis for the further diagnostic and treatment steps without an immediate or detailed check by the supervising physician.<br>Test and examination results are being reviewed and possibly checked by the supervising physician during the next regular meeting with the resident or ward round.
**Most relevant domains of competence**	(X) Medical Expert, () Communicator, () Collaborator, (X) Manager, () Health Advocate, () Scholar, (X) Professional
**Assessment sources**	Passing the assessment of the undergraduate medical programm and the final state examination. The assessment should involve knowledge testing, objective structured clinical examinations (including simulated patients), observations with patients in various clinical contexts (courses and clinical placements) and case-based discussions.
**Expected super-vision level at stage of training**	Distant supervision for entry into residency.

Title	Compile a treatment plan and initiate implementation (EPA 1.4)
	At the beginning of the postgraduate training, the resident is autonomously able to compile a structured treatment plan for adult patients with common diseases and typical cours of disease. Following coordination with the supervising physician, the resident initiates the implementation of the treatment plan.
**Specification**	The execution of the EPA includes:<br>1) requesting generic therapies (e.g. diet, fluid intake, regulating bowl activity, sleeping aides, simple pain medication),<br>2) compiling a treatment plan according to the test results, diagnoses and medication requirements of the patient,<br>3) presenting the plan to the supervising physician and achieving agreement.<br>Following the coordination with the supervising physician:<br>4) requesting patient-specific medication and therapy,<br>5) requesting basic therapy (e.g. physiotherapy, respiratory exercise),<br>6) sharing information with the patient and, where appropriate, family members,<br>7) sharing information with the wider care team (e.g. physicians, nurses).
**Limitations**	A closer supervision level than “act with distant supervision” is required for:<br>a) unstable or critically ill patients (e.g. patients in intensive care or emergency units),<br>b) new-borns, babies, children, adolescents or pregnant women,<br>c) discipline-specific clinical presentations or diseases (for example ophthalmology).
**Knowledge, skills and attitude**	Knowledge:<br>Pathophysiology of common diseases and their typical course; principles of prevention and therapy; standards of generic therapies; standards of established specific therapies for typical diseases; course, benefits, indications, contraindications and risks of common therapies (diet, substitution, physio- and ergotherapy, drugs, interventions, anesthesiology, surgery, radiation- and nucleotidtherapy); common drug interactions.<br>Skills:<br>Compiling a patient-specific, therapeutic plan; safe medication prescibing; consultation with the supervising physician; initiating patient-specific therapies; rational medical-decision making; informing patients and the care team.<br>Attitudes:<br>Attention to diversity (gender, age, culture); balancing effort, expense and risks of treatments against their benefits and results; dealing with uncertainty.
**Conditions and implications of entrustment decision**	The resident autonomously compiles a treatment plan. Following coordination with the supervising physician, he/ she requests common and/or patient-specific therapies. The resident initiates implementation without an immediate or detailed check by the supervising physician.<br>Implementation of the treatment plan will be reviewed and possibly checked by the supervising physician during the next regular meeting with the resident or ward round.
**Most relevant domains of competence**	(X) Medical Expert, () Communicator, (X) Collaborator, (X) Manager, () Health Advocate, (X) Scholar, () Professional
**Assessment sources**	Passing the assessment of the undergraduate medical programm and the final state examination. The assessment should involve knowledge testing, objective structured clinical examinations (including simulated patients), observations with patients in various clinical contexts (courses and clinical placements) and case-based discussions.
**Expected super-vision level at stage of training**	Distant supervision for entry into residency.

EPA domain 2 – General medical procedures

Title	**Perform general procedures of a physician (EPA 2)**
Specification	At the beginning of the postgraduate training, the resident is able to autonomously perform a defined set of general medical procedures.<br>The execution of this EPA includes:<br>1) explaining the procedure to the patient,<br>2) venous and capillary blood sampling (EPA 2.1 and 2.2),<br>3) inserting a peripheral catheter (EPA 2.3),<br>4) taking a blood culture (EPA 2.4),<br>5) taking a smear (oral, nasal, wound, anal, urogenital or cervical) (EPA 2.5),<br>6) giving an intracutaneous, subcutaneous or intramuscular injection (M. deltoideus) (EPA 2.6, 2.7 and 2.8),<br>7) giving an infusion (EPA 2.9),<br>8) placing a nasogastric tube (EPA 2.10),<br>9) performing a bed-site test to determine the blood group (EPA 2.11),<br>10) giving a blood product (e.g. erythrocytes, thrombocytes) (EPA 2.12),<br>11) putting on or changing a simple bandage (EPA 2.13),<br>12) taking an ECG (EPA 2.14),<br>13) writing a prescription (EPA 2.15).
Limitations	A closer supervision level than “act with distant supervision” is required for:<br>a) patients unable to give consent,<br>b) unstable or critically ill patients (i.e. patients in intensive care or emergency units),<br>c) newborns, babies, children, adolescents and pregnant women.
Knowledge, skills and attitude	Knowledge:<br>Structure and function of the human body relevant to the medical procedure; legal basis for performing the medical procedure; hygiene and infectious disease prevention; course, aims, indications, contraindications and potential risks of the medical procedure, prescribing medication.<br>Skills:<br>Preparing the procedure; securely identifying the patient; performing the medical procedure; post processing, including organising or excecuting the packing and distribution of patient-related materials; completing the requisition slip; writing a prescription.<br>Attitudes:<br>Putting the patient at ease; awareness of the risks involved for both the patient and oneself; use of language which is understandable to patients; attention to hygiene standards.
Conditions and implications of entrustment decision	The resident is able to perform the medical procedures autonomously with no immediately available supervising physician to assist.<br>Results of the procedure will be reviewed and possibly checked by the supervising physician during the next regular meeting with the resident or ward round.
Most relevant domains of competence	(X) Medical Expert, (X) Communicator, (X) Collaborator, (X) Manager, () Health Advocate, () Scholar, () Professional
Assessment sources	Passing the assessment of the undergraduate medical programm and the final state examination. The assessment should involve manikin-based simulation, objective structured clinical examinations (including simulated patients), observations with patients in various clinical contexts (courses and clinical placements) and case-based discussions.
Expected super-vision level at stage of training	Distant supervision for entry into residency.

EPA domain 3 – Communication with patients

**Title**	**Seek consent for medical examinations and procedures (EPA 3.1)**
**Specification**	At the beginning of the postgraduate training, the resident is able to autonomously inform patients about the course, benefits, risks and possible alternatives of a defined set of medical examinations and procedures and to seek patient consent.<br>The execution of this EPA includes:<br>1) explaining the medical examination and procedure to the patient,<br>2) filling in and completing the patient information sheet, obtaining the patient's signature and signing documents for the administration of erthocytes, thrombocytes or preparations of plasma,<br>3) Seeking oral patient consent for medical examinations and procedures which do not require written consent (i.e. taking blood, urinary catheter, feeding tubes, x-rays).
**Limitations**	A closer supervision level than "act with distant supervision" is required for:<br>a) patients unable to give consent,<br>b) medical examinations and procedures the resident is not familiar with,<br>c) unstable or critically ill patients (i.e. patients in intensive care or emergency units),<br>d) newborns, babies, children, adolescents and pregnant women,<br>e) surgical procedures, anaesthetics, and non-surgical interventions (e.g. angiography, angioplasty, stenting, fibrinolysis).
**Knowledge, skills and attitude**	<u>Knowledge:</u><br>Legal basis for performing medical examinations, procedures and interventions; patients' capacity to consent; course, aims, indications, contraindications and potential risks of medical examinations and medical procedures; principles and techniques of physician-patient communication and decision-making processes, risk communication as well as shared decision-making.<br><u>Skills:</u><br>Assessing capacity to consent; structuring the decision-making process; informing patients about the planned medical examination and procedure; communicating risks; seeking oral or written consent.<br><u>Attitudes:</u><br>Open and respectful communication; attention to diversity (gender, age, culture); use of language which is understandable to patients; dealing with uncertaintenty, compliance to confidentiality standards, respecting patient privacy.
**Conditions and implications of entrustment decision**	The patients´ consent sought by the resident forms the basis for the implementation of the medical examination or procedure without an immediate or detailed check by the supervising physician.<br>The consent will be reviewed and possibly checked by the supervising physician during the next regular meeting with the resident or ward round.
**Most relevant domains of competence**	() Medical Expert, (X) Communicator, () Collaborator, (X) Manager, () Health Advocate, () Scholar, (X) Professional
**Assessment sources**	Passing the assessment of the undergraduate medical programm and the final state examination. The assessment should involve objective structured clinical examinations (including simulated patients), observations with patients in various clinical contexts (courses and clinical placements) and case-based discussions.
**Expected supervision level at stage of training**	Distant supervision for entry into residency.

Title	**Inform and advise a patient (EPA 3.2)**
**Specification**	At the beginning of the postgraduate training, the resident is able autonomously to inform and advise adult patients who present with common diseases or counselling reasons.<br>The execution of this EPA includes:<br>1) gathering information concerning type of complaint and diagnosis of the patient,<br>2) providing general information about diagnostics, therapies and management,<br>3) following the consultation with the supervising physician, sharing and explaining information regarding the diagnostic and therapeutic steps to the patients, as well as the estimated duration of the treatment and hospital stay,<br>4) general advice on lifestyle changes (healthy diet, exercise, stress management),<br>5) general advice on alcohol and nicotine abuse,<br>6) general advice on sexually transmitted diseases.
**Limitations**	A closer supervision level than "act with distant supervision" is required for:<br>a) unstable or critically ill patients (i.e. patients in intensive care or emergency units),<br>b) breaking bad news (e.g. infuast prognosis),<br>c) domestic violence or emotionally challenging situations,<br>d) patients who are acute psychiatric or suicidal,<br>e) patients unable to give consent,<br>f) children and parents.
**Knowledge, skills and attitude**	<u>Knowledge</u><br>Principles of physician-patient communication, including active listening styles, considering content and relational aspects, the verbal and nonverbal level, as well as cognitive, emotional and motivational influencing factors; models and variables of health and illness, as well as prevention and health promotion; pathophysiology, standard diagnostic and therapy of typical clinical presentation medical results or common diseases.<br><u>Skills</u><br>Creating a trustful physician-patient relationship; realisation of a patient-centred conversation, consideration of the specific types, phases and purpose of the conversation; capture patients' health and lifestyle and working towards their improvement.<br><u>Attitudes</u><br>Empathy; open and respectful communication; attention to diversity (gender, age, culture); use of language which is understandable to patients; compliance to confidentiality standards, respecting patient privacy.
**Conditions and implications of entrustment decision**	The counselling carried out by the resident forms the basis for the diagnostic, therapy and management of the patient without an immediate or detailed check by the supervising physician.<br>Results of the patient counselling will be reviewed and possibly checked by the supervising physician during the next regular meeting with the resident or ward round.
**Most relevant domains of competence**	() Medical Expert, (X) Communicator, () Collaborator, () Manager, (X) Health Advocate, () Scholar, (X) Professional
**Assessment sources**	Passing the assessment of the undergraduate medical programm and the final state examination. The assessment should involve knowledge testing, objective structured clinical examinations (including simulated patients), observations with patients in various clinical contexts (courses and clinical placements) and case-based discussions.
**Expected supervision level at stage of training**	Distant supervision for entry into residency.

EPA domain 4 – Communication and collaboration with colleagues

Title	Present a patient history (EPA 4.1)
Specification	At the beginning of the postgraduate training, the resident is able to autonomously provide an oral report of a patient case in a structured manner, appropriate to the target audience and the situational requirements.<br><br>The execution of the EPA includes:<br>1) typical clinical presentations and common diseases with typical course,<br>2) non-typical clinical presentations and/or disease course and less common diseases following the consultation with the supervising physician,<br>3) a complete and focused presentation of the patient history according to the situational requirements, including current status and steps next to be taken,<br>4) presentation during ward rounds and patient-case discussions (e.g. clinical conference, x-ray demonstration, pathology conference, team meeting).
Limitations	A closer supervision level than “act with distant supervision” is required for:<br>a) unstable or critically ill patients (i.e. patients in intensive care or emergency unit),<br>b) newborns, babies, children, adolescents and pregnant women,<br>c) discipline-specific clinical presentations or diseases (for example ophthalmology).<br>d) presentations for surgery or invasive procedures,<br>e) histories of patients the resident does not personally care for.
Knowledge, skills and attitude	Knowledge:<br>Clinical presentations, pathophysiology and test results of typical clinical presentations or common diseases; structure of a complete or focussed presentation of a patient history; aims and course of ward rounds and patient-specific case discussions.<br>Skills:<br>Presenting a patient history appropriate to the audience and the context, including differentiation between main and additional findings, diagnoses, suspected or working diagnoses; addressing open questions and if necessary seeking advice or decisions on the further management of the patient.<br>Attitudes:<br>Open communication; attention to diversity (gender, age, culture); attention to hygiene standards; compliance to confidentiality standards, respecting patient privacy.
Conditions and implications of entrustment decision	The presentation of the patient history through the resident forms the basis for the further management of the patient within the care team without an immediate or detailed check by the supervising physician.<br>Patient history, test results and entries into the patient file will be reviewed and possibly checked by the supervising physician during the next regular meeting with the resident or ward round.
Most relevant domains of competence	(X) Medical Expert, (X) Communicator, (X) Collaborator, () Manager, () Health Advocate, () Scholar, (X) Professional
Assessment sources	Passing the assessment of the undergraduate medical programm and the final state examination. The assessment should involve objective structured clinical examinations (including simulated patients) and observations in various clinical contexts (courses and clinical placements).
Expected supervision level at stage of training	Distant supervision for entry into residency.

Title	Give or receive a patient handover (EPA 4.2)
Specification	At the beginning of the postgraduate training, the resident is able to autonomously give or receive a structured patient handover for adult patients to and from other medical personnel or departments, appropriate to the target audience and the situational requirements.<br>The execution of the EPA includes:<br>1) common diseases with typical course,<br>2) less common diseases and/or disease courses following the consultation with the supervising physician,<br>3) a complete and focused handover of the patient history according to the situational requirements including current status and steps next to be taken<br>4) shift change or patient handover to/ from physicians in the same clinical department,<br>5) patient handover to/from non-medical staff (e.g. nursing, other caregivers, patient transport).
Limitations	A closer supervision level than “act with distant supervision” is required for:<br>a) unstable or critically ill patients (i.e. patients in intensive care or emergency units),<br>b) handovers to/from surgery and invasive interventions,<br>c) handovers to/from intensive care and emergency units,<br>d) newborns, babies, children, adolescents and pregnant women,<br>e) discipline-specific clinical presentations or diseases (for example ophthalmology),<br>f) patients the resident does not personally care for.
Knowledge, skills and attitude	Knowledge:<br>Clinical presentation, pathophysiology and findings of typical clinical presentations or common diseases; structure of the patient history according to the aim and course of the handover; principles of oral, written and electronic communication, as well as medical confidentiality and data protection.<br>Skills:<br>Presentation of a patient history according to the aim and setting of the handover, including differentiation of main and additional findings, diagnoses and suspected diagnosis; addressing open questions, as well as problems or tasks which may arise after the handover.<br>Attitudes:<br>Open communication; attention to diversity (gender, age, culture); compliance to confidentiality standards, respecting patient privacy.
Conditions and implications of entrustment decision	The patient handover carried out by the resident forms the basis of further diagnostics, therapies and management of the patient without an immediate or detailed check by the supervising physician.<br>Outcome of the patient handover will be reviewed and possibly checked by the supervising physician during the next regular meeting with the resident or ward round.
Most relevant domains of competence	(X) Medical Expert, (X) Communicator, (X) Collaborator, (X) Manager, () Health Advocate, () Scholar, () Professional
Assessment sources	Passing the assessment of the undergraduate medical programm and the final state examination. The assessment should involve objective structured clinical examinations (including simulated patients) and observations in various clinical contexts (courses and clinical placements).
Expected super-vision level at stage of training	Distant supervision for entry into residency.

Title	Write and distribute a patient report (EPA 4.3)
Specification	At the beginning of the postgraduate training, the resident is able to autonomously write a structured patient report and distribute to it to appropriate personnel or departments.<br>The execution of the EPA includes:<br>1) writing and signing of preliminary discharge, transfer or interim reports based on the consultation with the supervising physician on the further management of the patient,<br>2) submitting, revising and counter-signing of the final report by the supervising physician,<br>3) structuring the report in line with local specifications (i.e. departmental letter head, patient identifikation, list of diagnoses, medical history and examination, results, summary and patients´course, medication at discharge,<br>4) distributing the report to the appropriate personnel and departments, if necessary initiate accordingly.
Limitations	A closer supervision level than “act with distant supervision” is required for:<br>a) transfer to the intensive care unit,<br>b) patients the resident does not personally care for.
Knowledge, skills and attitude	Knowledge:<br>Clinical presentation, pathophysiology and findings of typical clinical presentations and common diseases; structure of a written patient report tailored to the target group; principles of oral, written and electronic communication, as well as medical confidentiality and data protection.<br>Skills:<br>Preparation of a written patient case in form of a discharge, transfer, and interim report; transmission in provisional form; coordination with the supervising physician; preparation and transmission of the final report.<br>Attitudes:<br>Communication targeted to the audience; attention to diversity (gender, age, culture); compliance to confidentiality standards, respecting patient privacy.
Conditions and implications of entrustment decision	The interim report prepared by the resident forms the basis for the further diagnostic, therapy and management of the patient without an immediate or detailed check by the supervising physician. The final report will be prepared in coordination with the supervising physician.<br>The transmission of the final report to the appropriate personnel or departments will be carried out by the resident without an immediate or detailed check by the supervising physician.
Most relevant domains of competence	(X) Medical Expert, (X) Communicator, (X) Collaborator, (X) Manager, () Health Advocate, () Scholar, () Professional
Assessment sources	Passing the assessment of the undergraduate medical programm and the final state examination. The assessment should involve objective structured clinical examinations (including simulated patients) and observations in various clinical contexts (courses and clinical placements).
Expected super-vision level at stage of training	Distant supervision for entry into residency.

EPA domain 5 – Patient care in special situations

Title	Recognise an emergency situation and act upon it (EPA 5.1)
Specification	At the beginning of the postgraduate training, the resident is able to autonomously recognise an emergency situation and to broadly assess its scope. The resident is able to autonomously provide emergency aid and to call for help for the further management of the patient.<br>The execution of the EPA includes:<br>1) calling for help according to the local circumstances (e.g. emergency team),<br>2) beginning basic-life support without technical resources in patients of any age showing loss of vital functions,<br>3) beginning reanimation with technical resources (e.g. ambu breathing bag; defibrillator) in adult patients showing loss of vital functions,<br>4) recognising situations of imminent vital threat and start managing the patient (sign for shortness of breath or hypoxia, chest pain, increasing impairment of consciousness, high temperature, arterial hypo- and hypertension, tachy- and bradycardia, hypo- and hyperglycaemia, anuria, internal and external bleeding, trauma and injury),<br>5) immediately informing the supervising physician and requesting support.
Limitations	A closer supervision level than "act with distant supervision" is required for:<br>a) reanimation of non-adult patients with technical resources,<br>b) performing the continuing management in conditions of vital threat.
Knowledge, skills and attitude	Knowledge:<br>Rescue chain in and outside the hospital; manifestation, pathophysiology, standard diagnostic and therapy in conditions of imminent vital threat; basic and advanced life support; airway management, trauma management.<br>Skills:<br>Recognising and acting on conditions of imminent vital threat; initiation of a rescue chain; performing basic and advanced life support; handling the defibrillator; positioning of emergency patients; working in a team to manage an emergency patient.<br>Attitudes:<br>Recognising the role as first aider; preserving the safety of the patient and one's own.
Conditions and implications of entrustment decision	The resident immediately requests support for patients with loss of vital functions and conditions of vital threat. He/ she provides emergency aid until the arrival of the emergency team or the supervising physician.<br>The emergency situation and the procedure will be discussed in retrospect with the supervising physician.
Most relevant domains of competence	(X) Medical Expert, (X) Communicator, (X) Collaborator, (X) Manager, () Health Advocate, () Scholar, () Professional
Assessment sources	Passing the assessment of the undergraduate medical programm and the final state examination. The assessment should involve knowledge testing, manikin-based simulation and objective structured clinical examinations (including simulated patients).
Expected supervision level at stage of training	Distant supervision for entry into residency.

**Title**	**Undertake an evidence-based patient case presentation and initiate patient-specific implementation (EPA 5.2)**
**Specification**	At the beginning of the postgraduate training, the resident is able to autonomously collate best available evidence concerning a medical problem of an individual patient and to present it in a structured manner. The resident is able to initiate adjustments to the management of the patient following coordination with the supervising physician or physician team.<br>The execution of this EPA includes:<br>1) medical problems which can be handled by the resident,<br>2) searching for best available evidence (using data bases and scientific journals), including guidelines and clinical reviews,<br>3) checking the clinical relevance and applicability for the individual patient,<br>4) delivering a structured patient case presentation (i.e. departmental discussion, in-house training session),<br>5) seeking agreement with the supervising physician in terms of what changes should be applied to the management of the patient,<br>6) requesting changes according in line with EPA 1.2 ”diagnostic plan” and EPA 1.4 “treatment plan”.
**Limitations**	A closer supervision level than “act with distant supervision” is required for:<br>a) presentations of a medical problem which are too complex,<br>b) presentations of a medical problem with little clinical evidence,<br>c) evidence which primarily relies on basic research.
**Knowledge, skills and attitude**	Knowledge:<br>Principles of medical problem definition and evidence-based medicine; methodology, use and limitations of evidence-based medicine, including formulation of the search question, literature search and appraisal as well as judging its patient-specific applicability, course and structure of a patient case presentation.<br>Skills:<br>Searching for and grading evidence-based literature for an individual patient case and medical problem; presenting the case to the team; making adjustments, seeking agreement with the supervising physician and initiate implementation of a patient-specific management plan; rational medical decision-making; informing patients and care team.<br>Attitudes:<br>Attention to diversity (gender, age, and culture); balancing effort, expense and risks of diagnostics and therapies against benefits and results; dealing with diagnostic uncertainty.
**Conditions and implications of entrustment decision**	The resident presents the results of the evidence search and its application to the individual patient to the physicians’ team. Following coordination with the supervising physician, he/ she adjusts the management plan of the patient and initiates implementation without an immediate or detailed check by the supervising physician. Implementation of the adjusted management plan will be reviewed and possibly checked by the supervising clinician during the next regular meeting with the resident or ward round.
**Most relevant domains of competence**	(X) Medical Expert, () Communicator, () Collaborator, (X) Manager () Health Advocate, (X) Scholar, () Professional
**Assessment sources**	Passing the assessment of the undergraduate medical programm and the final state examination. The assessment should involve knowledge testing as well as evidence-based case elaboration and presentations in various clinical contexts (courses and clinical placements).
**Expected super-vision level at stage of training**	Distant supervision for entry into residency.
